# Post-Transcriptional Control of Type I Interferon Induction by Porcine Reproductive and Respiratory Syndrome Virus in Its Natural Host Cells

**DOI:** 10.3390/v4050725

**Published:** 2012-05-02

**Authors:** Xiuqing Wang, Jane Christopher-Hennings

**Affiliations:** 1 Department of Biology and Microbiology, South Dakota State University, Brookings, SD 57007, USA; 2 Department of Veterinary and Biomedical Sciences, South Dakota State University, Brookings, SD 57007, USA; Email: jane.hennings@sdstate.edu

**Keywords:** PRRSV, Type I interferon, post-transcriptional regulation, translational control

## Abstract

Porcine reproductive and respiratory syndrome virus (PRRSV) is not only a poor inducer of type I interferon but also inhibits the efficient induction of type I interferon by porcine transmissible gastroenteritis virus (TGEV) and synthetic dsRNA molecules, Poly I:C. However, the mechanistic basis by which PRRSV interferes with the induction of type I interferon in its natural host cells remains less well defined. The purposes of this review are to summarize the key findings in supporting the post-transcriptional control of type I interferon in its natural host cells and to propose the possible role of translational control in the regulation of type I interferon induction by PRRSV.

## 1. Introduction

Porcine reproductive and respiratory syndrome virus (PRRSV) is a single-stranded, positive-sense RNA virus with a genome size of approximately 15 kb. PRRSV belongs to the *Arteriviridae* in the order of *Nidovirale*. PRRSV causes acute respiratory disease in neonatal and young piglets and reproductive failure in pregnant sows. PRRSV primarily infects and destroys alveolar macrophages during acute infection of swine. In addition to macrophages, PRRSV has also been identified by immunohistochemistry in dendritic-like cells in tonsils and lymph nodes [1], suggesting that dendritic cells may either be susceptible to PRRSV or capture PRRSV by taking up apoptotic infected cells. Recent studies have shown that porcine monocyte-derived dendritic cells are highly susceptible to PRRSV infection *in vitro* [2,3]. In contrast, both lung-derived dendritic cells and plasmacytoid dendritic cells are resistant to PRRSV infection *in vitro* [3,4]. The role of dendritic cells in PRRSV infection *in vivo* remains to be determined. Similar to other members of the *Arteriviridae,* one of the hallmark features of PRRSV infection is its persistence in the host, which makes the control and elimination of the disease difficult to achieve. There are two major genotypes of PRRSV, North American type (or type II) and European type (or type I). PRRSV is currently distributed worldwide and causes significant economic losses to the swine industry [5].

Type I interferon including interferon-α and β is one of the most important innate defense mechanisms of the host against virus infections [6]. Virtually all cell types such as epithelial cells, fibroblasts, macrophages, and dendritic cells are capable of producing type I interferon when they are exposed to viruses or other interferon inducing stimuli. However, interferon-α is preferably induced by leukocytes such as macrophages and dendritic cells. Most RNA viruses induce type I interferon by sensing through the endosomal toll-like receptors 3 and 7 (TLR3, 7), the cytoplasmic retinoic acid-inducible gene I (RIG-I), and/or melanoma differentiation-associated gene-5 (MDA-5) [7,8,9]. Both TLR3 and RIG-1 recognize dsRNA molecules generated during RNA virus infections, but they interact with different adaptor proteins to activate kinases TBK1 (tank-binding kinase 1) and IKK (IkappaB kinase). TLR3 interacts with TRIF (TIR-domain-containing adapter-inducing interferon-β), a TLR adaptor molecule. While the amino-terminal CARD (caspase-recruiting domain) [10] of RIG-I interacts with a second CARD containing protein, IPS (interferon-β stimulator)-1, after dsRNA binds to the carboxyl terminus of RIG-I, to activate the kinases TBK1 and IKK [7,10]. Activation of TBK1 and IKK leads to the activation and phosphorylation of interferon regulatory factor 3 (IRF-3). Phosphorylated IRF 3 translocates to the nucleus and binds to the DNA elements to activate the transcription of interferon-α and β [7,10]. In addition to IRF-3, interferon regulatory factor -7 (IRF-7) also plays some role in the late phase of interferon-α and β induction [11]. Sun *et al*. have provided a more detailed description on the signaling pathways for type I interferon production [12].

More recently, studies have suggested the important role of IRF-7 in interferon-α induction from plasmacytoid dendritic cells [13,14]. The phosphatidylinositol-3 kinase (PI3K) pathway seems to be critical to the nuclear translocation of IRF-7 and the interferon-α induction. A recent study suggested that TLR triggering by heat-inactivated influenza A virus (TLR7 ligand) and CpG oligodeoxynucleotide (ODN) (TLR9 ligand) leads to PI3K activation. The transcription and translation of type I interferon including interferon-α and β, but not other inflammatory cytokines such as interleukin-6 (IL-6) and tumor necrosis factor-α (TNF-α), are absolutely dependent on activation of PI3K [9]. Although the nuclear factor kappa B (NF-κB) pathway has been shown to contribute to the transcriptional activation of type I interferon by PRRSV [15], one study shows that NF-κB is more likely related to induction of inflammatory cytokines such as TNF-α and IL-6, rather than type I interferon, after influenza A virus infection and CpG ODN stimulation [9]. This discrepancy may be due to the cell types and viruses used in different studies since different cell types and different viruses exhibit distinct features in the induction of type I interferon pathway. Virus replication and viral infectivity are usually not essential to the induction of type I interferon since both UV-inactivated and heat-inactivated influenza A viruses induce more abundant interferon-α than their live virus counterparts [16].

## 2. Transcriptional Control of Type I Interferon by PRRSV

In 1998, Albina *et al*. first reported the failure of PRRSV in inducing the production of interferon-α protein in the lung secretions of infected pigs and in the supernatants of PRRSV-infected alveolar macrophages and peripheral blood mononuclear cells [17]. Furthermore, they observed that PRRSV was also capable of blocking the production of interferon-α protein in macrophages by a well-characterized and potent interferon-α inducer, swine transmissible gastroenteritis virus (TGEV), a member of the *coronaviridae* family*.* Virus replication and infectivity is essential to the inhibition of interferon-α production since UV-inactivated PRRSV fails to block the induction of interferon-α by TGEV. Interestingly, a recent study reported that PRRSV infectivity is not essential to the inhibition of type I interferon in plasmacytoid dendritic cells, which are resistant to PRRSV infection [4]. The observation that PRRSV is a poor inducer of interferon-α is further confirmed by Buddaert *et al*. [18]. In 2004, Chung *et al*. reported that PRRSV activated the transcription of interferon-α and Mx1 in lung tissues at day 1 and peaked at day 7 after infection in acutely infected animals, suggesting that PRRSV does activate the transcription of interferon-α and interferon induced genes such as Mx1 [19]. Others have further reported that different PRRSV isolates exhibit different capacities in inducing the production of interferon-α in alveolar macrophages [20]. The activation of interferon-β transcription in PRRSV-infected monocyte-derived dendritic cells, peripheral blood mononuclear cells, alveolar macrophages and in porcine alveolar macrophages of PRRSV-infected animals has also been reported [3,21,22,23]. Taken together, the existing evidence clearly indicates that PRRSV activates the transcription of type I interferon in its natural susceptible cells. In MARC-145 cells, however, Miller reported that PRRSV not only failed to activate the transcription of interferon-α and β, but also suppressed the transcription of interferon-β activated by Poly I:C [24]. This suggests a cell type dependent difference in activating the transcription of type I interferon by PRRSV. Marked differences in type I interferon induced antiviral activity against PRRSV between porcine alveolar macrophages and MARC-145 cells have also been reported recently [25]. Since MARC-145 cells are not the natural susceptible cells for PRRSV, the implication of the results in understanding the pathogenesis of PRRSV is debatable. Overall, the existing evidence clearly suggests that PRRSV may have intrinsic properties to inhibit or reduce the induction of interferon-α and β.

To further elucidate the molecular mechanisms by which PRRSV inhibits the induction of interferon-β transcription in MARC-145 cells, Luo *et al*. examined the role of several key molecules in mediating the transcriptional activation of interferon-β [26]. They reported that PRRSV interferes with the nuclear translocalization of IRF-3 by inactivating IPS-1, a downstream molecule of the RIG-I pathway [26]. Studies by Beura *et al*., suggested that the nonstructural proteins of PRRSV, including Nsp1, Nsp2, Nsp11 and Nsp4, when over-expressed in cell culture alone have the ability to antagonize the nuclear translocation of IRF-3 and the promoter activity of interferon-β activated by Poly I:C and Sendai virus [27]. Similar studies have to some degree confirmed that over-expressed Nsp1 and Nsp2 of PRRSV in cell culture antagonize the promoter activity of interferon-β as determined by using the luciferase reporter gene expression system [15,28,29,30]. Sun *et al*. provided a detailed review on the role of PRRSV nonstructural and structural proteins in modulating the transcriptional activation of type I interferon in MARC-145 cells and human cell culture system including HeLa cells and 293T cells [12]. Despite the ease and convenience of using the luciferase reporter system to dissect the role of over-expressed viral proteins in regulating the transcription of interferon-β, such results are often contradictory to the authentic virus infection of natural susceptible cells *in vitro* and *in vivo* [10,16,31]. Therefore, the implication of such results in natural virus infection is uncertain.

## 3. Post-Transcriptional and Translational Control of Type I Interferon by PRRSV

In 2004, Lee *et al*. first described the discrepancy between interferon-α mRNA level and interferon-α protein production in PRRSV-infected porcine alveolar macrophages [20]. They suggested that a post-transcriptional regulatory mechanism contributed to the inhibition of interferon-α production. We have observed the same phenomenon in PRRSV-infected porcine monocyte-derived dendritic cells [32]. Despite the transient and abundant interferon-α and β mRNA molecules, very little or no interferon-α protein was detected in either cell lysates or supernatants of PRRSV-infected cells at different time points after virus infection, indicating a post-transcriptional control of type I interferon induction. Currently, very little is known about the translational control of type I interferon production by PRRSV. PRRSV inhibits the PI3K-dependent Akt (PI3K/Akt) pathway during late infection [33], which makes the translation repressor, 4E-BP1, hyperactive and reduces global protein synthesis. Interestingly, a recent study has demonstrated that PI3K/Akt inhibition can also lead to the phosphorylation of eIF-2α to inhibit cellular translation [34]. We have indeed observed increased phosphorylation of eIF-2α in PRRSV-infected porcine Mo-DC during late infection (unpublished observation). Thus, it is possible that the translation of IFN-α is reduced partly by PI3K/Akt inhibition. However, it is more likely that PRRSV has employed multiple strategies to inhibit cellular protein synthesis as a simple way to evade the host defenses, which may also contribute to the inhibited type I interferon induction. In response to some virus infections, host dsRNA-activated protein kinase (PKR) phosphorylates eIF-2α to inhibit both cellular and viral translation [35,36,37]. PRRSV replication is known to trigger the stress-activated proteins kinases to modulate cytokine production in porcine alveolar macrophages [38]. It has also been demonstrated that cleavage of the eukaryotic translation initiation factor 4G (eIF4G) by either viral proteases or cellular proteases such as caspase 3 activated during apoptosis can result in rapid block of cellular protein synthesis [39,40,41,42]. Furthermore, poly(A) tail elongation mediated by viruses may repress the efficient translation of type I interferon [43].

Both UV-inactivated and heat-inactivated influenza A viruses are more potent than wild-type viruses in inducing the production of type I interferon [16]. Cytopathogenecity has been associated with translational shut-down of host genes including interferon [44]. PRRSV is highly pathogenic in alveolar macrophages and monocyte-derived dendritic cells and rapidly destroys these target cells by apoptosis and necrosis [2,45,46]. Studies have clearly shown that interferon mRNA has a short life span after induction [43,47]. We speculate that the combination of high cytopathogenicity of virus and short half-life of interferon mRNAs may at least partially contribute to the low interferon proteins detected in virus-infected cells. It is also possible that the variability of different PRRSV isolates in inducing type I interferon is related to their varied cytopathogenicity [20,23,48]. For influenza A virus, different isolates can vary in their ability to induce interferon by up to 100-fold [16]. Finally, it remains to be determined whether PRRSV induces the shutoff of host protein synthesis to favor its own protein synthesis. More research efforts should be directed to the understanding of the translational control of type I interferon by PRRSV in its natural host cells.

## 4. Conclusions

PRRSV activates the transcription of type I interferon in porcine alveolar macrophages, peripheral blood mononuclear cells, and monocyte-derived dendritic cells. However, PRRSV interferes with the translation of type I interferon in these cells partly through cytopathogenicity since UV and heat-inactivated viruses lose their ability to interfere with the induction of type I interferon by porcine transmissible gastroenteritis virus or Poly I:C. Further studies are needed to delineate the exact mechanisms by which PRRSV interferes with the translation of type I interferon in its natural host cells.
